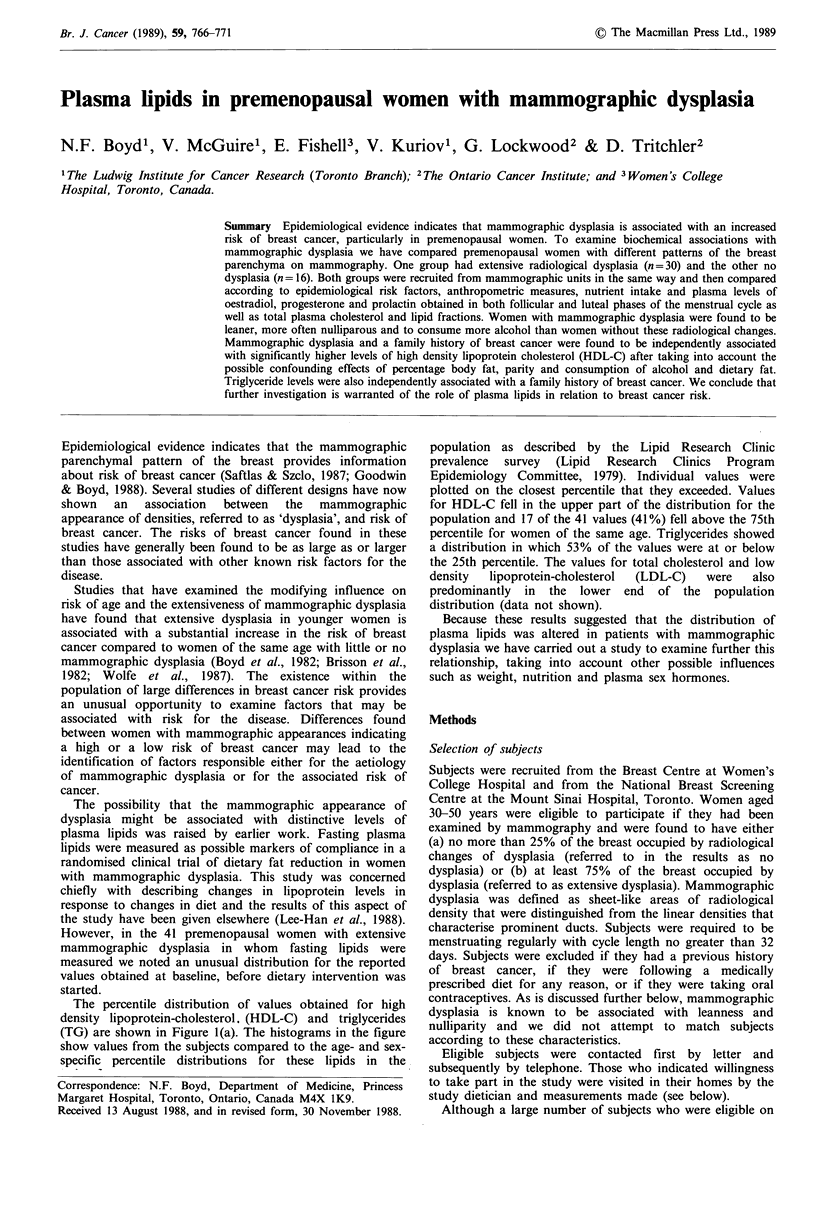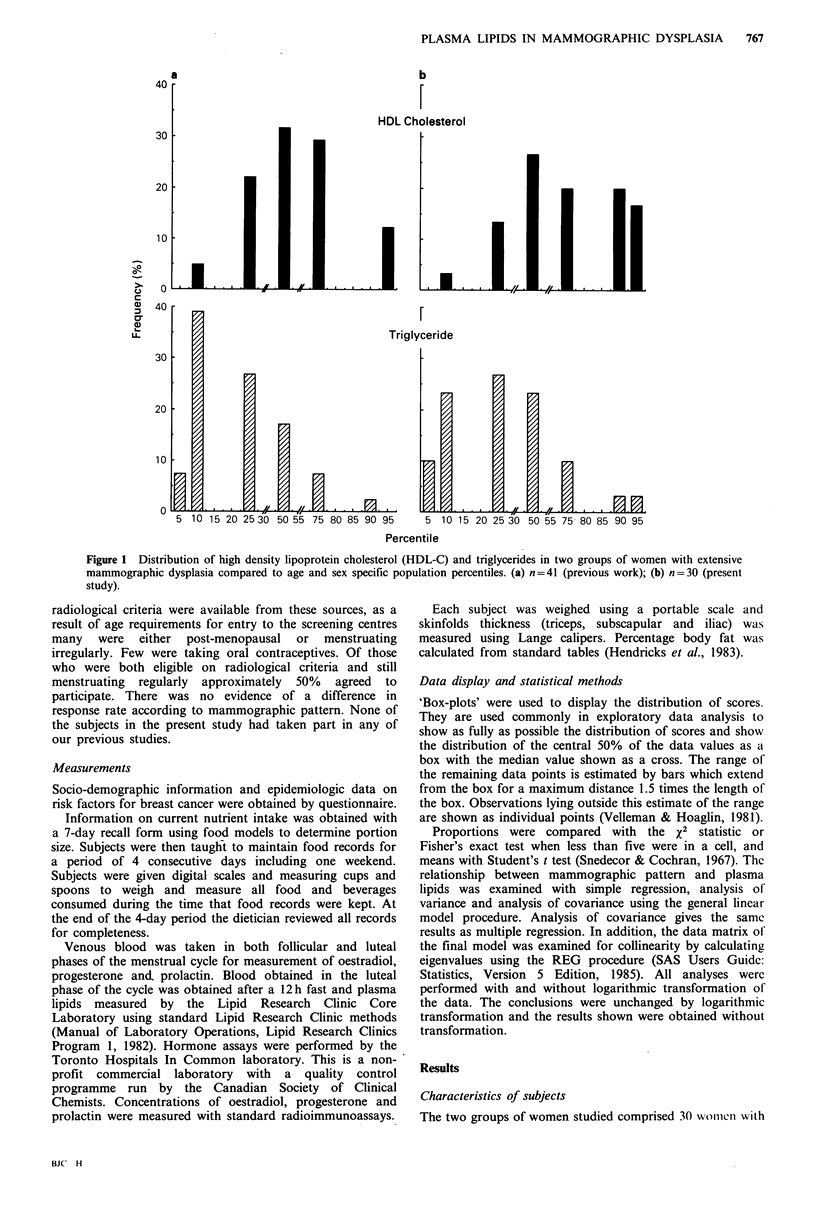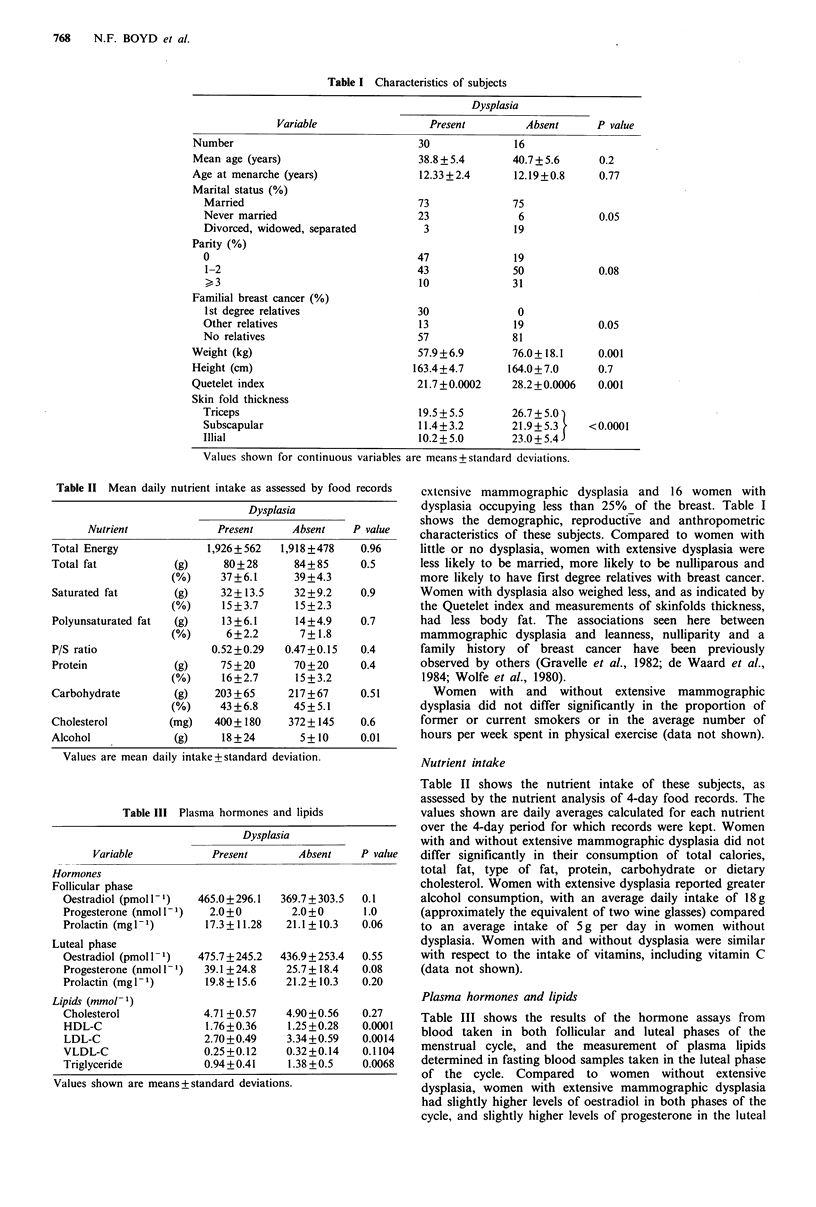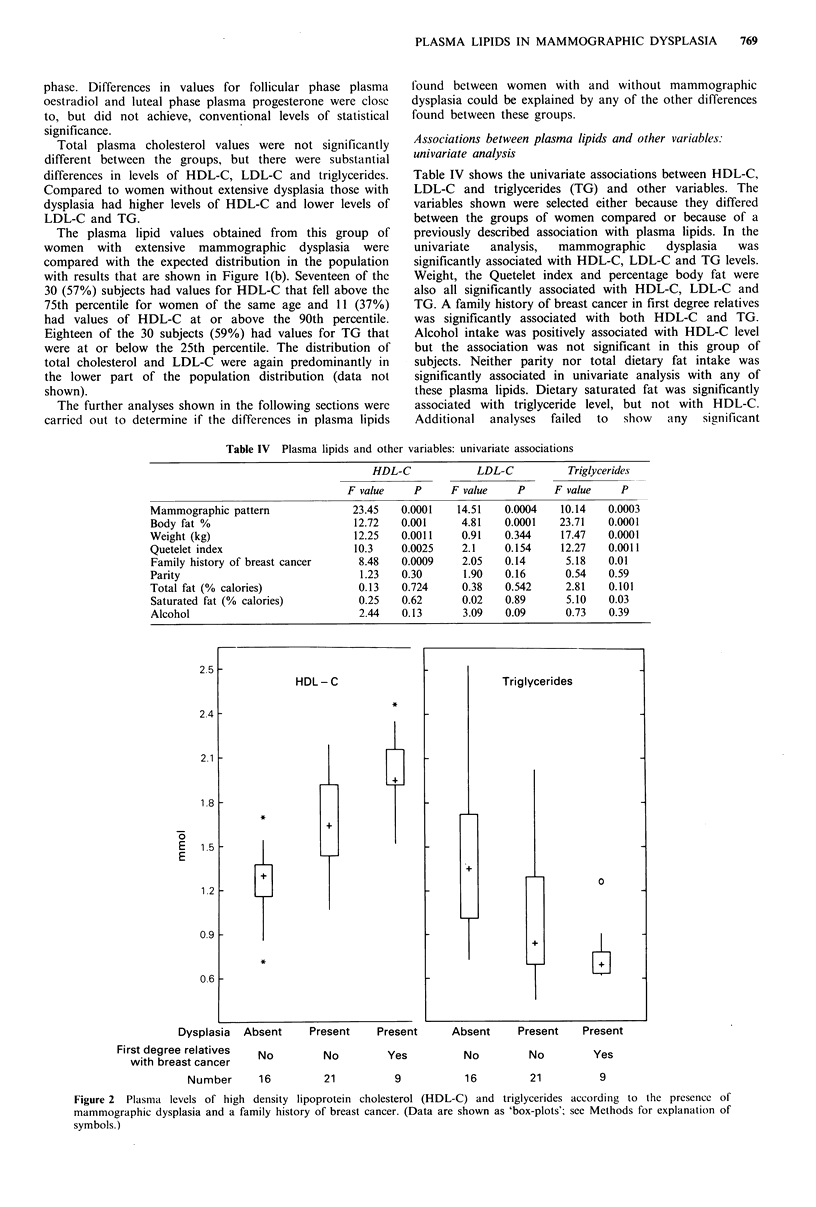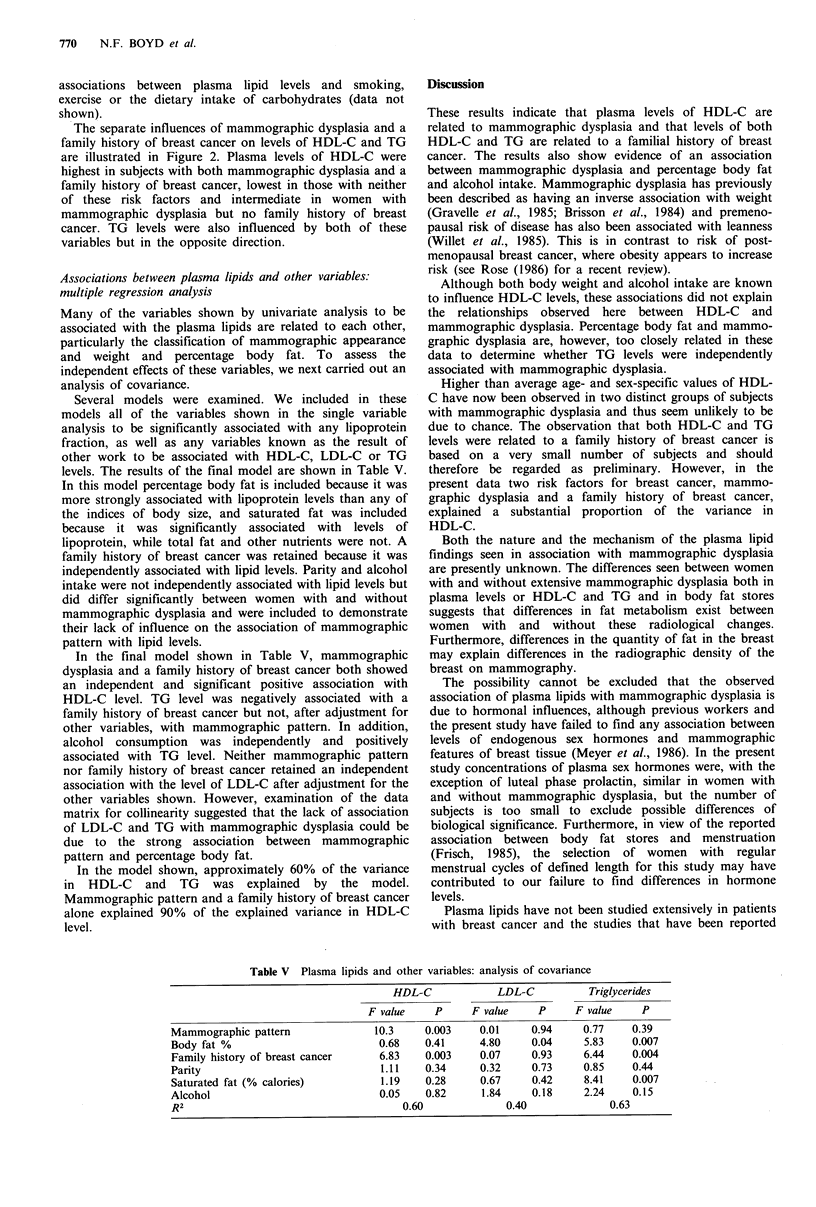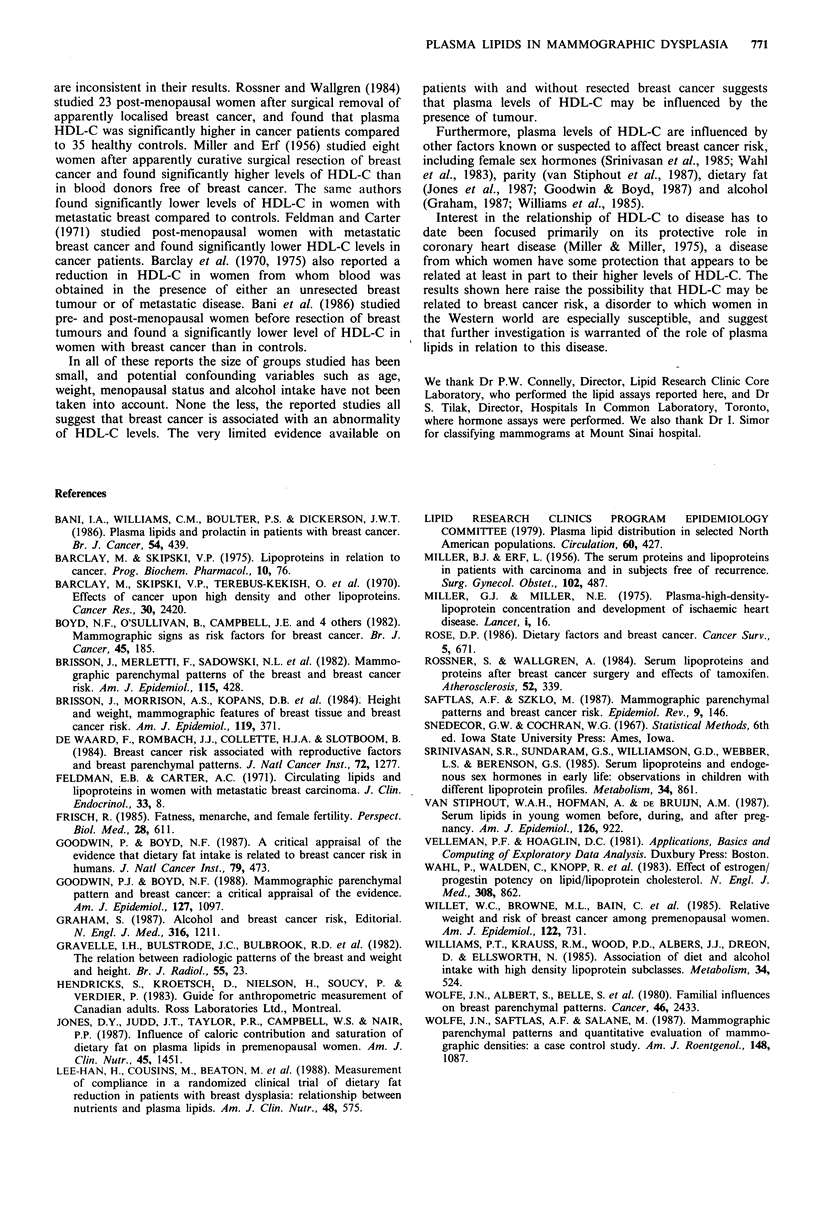# Plasma lipids in premenopausal women with mammographic dysplasia.

**DOI:** 10.1038/bjc.1989.160

**Published:** 1989-05

**Authors:** N. F. Boyd, V. McGuire, E. Fishell, V. Kuriov, G. Lockwood, D. Tritchler

**Affiliations:** Ludwig Institute for Cancer Research (Toronto Branch), Canada.

## Abstract

Epidemiological evidence indicates that mammographic dysplasia is associated with an increased risk of breast cancer, particularly in premenopausal women. To examine biochemical associations with mammographic dysplasia we have compared premenopausal women with different patterns of the breast parenchyma on mammography. One group had extensive radiological dysplasia (n = 30) and the other no dysplasia (n = 16). Both groups were recruited from mammographic units in the same way and then compared according to epidemiological risk factors, anthropometric measures, nutrient intake and plasma levels of oestradiol, progesterone and prolactin obtained in both follicular and luteal phases of the menstrual cycle as well as total plasma cholesterol and lipid fractions. Women with mammographic dysplasia were found to be leaner, more often nulliparous and to consume more alcohol than women without these radiological changes. Mammographic dysplasia and a family history of breast cancer were found to be independently associated with significantly higher levels of high density lipoprotein cholesterol (HDL-C) after taking into account the possible confounding effects of percentage body fat, parity and consumption of alcohol and dietary fat. Triglyceride levels were also independently associated with a family history of breast cancer. We conclude that further investigation is warranted of the role of plasma lipids in relation to breast cancer risk.


Br. J. Cancer (1989), 59, 766–771 © The Macmillan Press Ltd., 1989

### Plasma lipids in premenopausal women with mammographic dysplasia

N.F. Boyd[1], V. McGuire[1], E. Fishell[3], V. Kuriov[1], G. Lockwood[2] & D. Tritchler[2]

[1]*The Ludwig Institute for Cancer Research (Toronto Branch);* [2]*The Ontario Cancer Institute; and* [3]*Women's College Hospital, Toronto, Canada.*

**Summary** Epidemiological evidence indicates that mammographic dysplasia is associated with an increased risk of breast cancer, particularly in premenopausal women. To examine biochemical associations with mammographic dysplasia we have compared premenopausal women with different patterns of the breast parenchyma on mammography. One group had extensive radiological dysplasia ($n=30$) and the other no dysplasia ($n=16$). Both groups were recruited from mammographic units in the same way and then compared according to epidemiological risk factors, anthropometric measures, nutrient intake and plasma levels of oestradiol, progesterone and prolactin obtained in both follicular and luteal phases of the menstrual cycle as well as total plasma cholesterol and lipid fractions. Women with mammographic dysplasia were found to be leaner, more often nulliparous and to consume more alcohol than women without these radiological changes. Mammographic dysplasia and a family history of breast cancer were found to be independently associated with significantly higher levels of high density lipoprotein cholesterol (HDL-C) after taking into account the possible confounding effects of percentage body fat, parity and consumption of alcohol and dietary fat. Triglyceride levels were also independently associated with a family history of breast cancer. We conclude that further investigation is warranted of the role of plasma lipids in relation to breast cancer risk.

Epidemiological evidence indicates that the mammographic parenchymal pattern of the breast provides information about risk of breast cancer (Saftlas & Szclo, 1987; Goodwin & Boyd, 1988). Several studies of different designs have now shown an association between the mammographic appearance of densities, referred to as 'dysplasia', and risk of breast cancer. The risks of breast cancer found in these studies have generally been found to be as large as or larger than those associated with other known risk factors for the disease.

Studies that have examined the modifying influence on risk of age and the extensiveness of mammographic dysplasia have found that extensive dysplasia in younger women is associated with a substantial increase in the risk of breast cancer compared to women of the same age with little or no mammographic dysplasia (Boyd *et al.*, 1982; Brisson *et al.*, 1982; Wolfe *et al.*, 1987). The existence within the population of large differences in breast cancer risk provides an unusual opportunity to examine factors that may be associated with risk for the disease. Differences found between women with mammographic appearances indicating a high or a low risk of breast cancer may lead to the identification of factors responsible either for the aetiology of mammographic dysplasia or for the associated risk of cancer.

The possibility that the mammographic appearance of dysplasia might be associated with distinctive levels of plasma lipids was raised by earlier work. Fasting plasma lipids were measured as possible markers of compliance in a randomised clinical trial of dietary fat reduction in women with mammographic dysplasia. This study was concerned chiefly with describing changes in lipoprotein levels in response to changes in diet and the results of this aspect of the study have been given elsewhere (Lee-Han *et al.*, 1988). However, in the 41 premenopausal women with extensive mammographic dysplasia in whom fasting lipids were measured we noted an unusual distribution for the reported values obtained at baseline, before dietary intervention was started.

The percentile distribution of values obtained for high density lipoprotein-cholesterol, (HDL-C) and triglycerides (TG) are shown in Figure 1(a). The histograms in the figure show values from the subjects compared to the age- and sex-specific percentile distributions for these lipids in the population as described by the Lipid Research Clinic prevalence survey (Lipid Research Clinics Program Epidemiology Committee, 1979). Individual values were plotted on the closest percentile that they exceeded. Values for HDL-C fell in the upper part of the distribution for the population and 17 of the 41 values (41%) fell above the 75th percentile for women of the same age. Triglycerides showed a distribution in which 53% of the values were at or below the 25th percentile. The values for total cholesterol and low density lipoprotein-cholesterol (LDL-C) were also predominantly in the lower end of the population distribution (data not shown).

Because these results suggested that the distribution of plasma lipids was altered in patients with mammographic dysplasia we have carried out a study to examine further this relationship, taking into account other possible influences such as weight, nutrition and plasma sex hormones.

### Methods

### *Selection of subjects*

Subjects were recruited from the Breast Centre at Women's College Hospital and from the National Breast Screening Centre at the Mount Sinai Hospital, Toronto. Women aged 30–50 years were eligible to participate if they had been examined by mammography and were found to have either (a) no more than 25% of the breast occupied by radiological changes of dysplasia (referred to in the results as no dysplasia) or (b) at least 75% of the breast occupied by dysplasia (referred to as extensive dysplasia). Mammographic dysplasia was defined as sheet-like areas of radiological density that were distinguished from the linear densities that characterise prominent ducts. Subjects were required to be menstruating regularly with cycle length no greater than 32 days. Subjects were excluded if they had a previous history of breast cancer, if they were following a medically prescribed diet for any reason, or if they were taking oral contraceptives. As is discussed further below, mammographic dysplasia is known to be associated with leanness and nulliparity and we did not attempt to match subjects according to these characteristics.

Eligible subjects were contacted first by letter and subsequently by telephone. Those who indicated willingness to take part in the study were visited in their homes by the study dietician and measurements made (see below).

Although a large number of subjects who were eligible on

Correspondence: N.F. Boyd, Department of Medicine, Princess Margaret Hospital, Toronto, Ontario, Canada M4X 1K9.
Received 13 August 1988, and in revised form, 30 November 1988.

**Figure 1** Distribution of high density lipoprotein cholesterol (HDL-C) and triglycerides in two groups of women with extensive mammographic dysplasia compared to age and sex specific population percentiles. (**a**) $n=41$ (previous work); (**b**) $n=30$ (present study).

radiological criteria were available from these sources, as a result of age requirements for entry to the screening centres many were either post-menopausal or menstruating irregularly. Few were taking oral contraceptives. Of those who were both eligible on radiological criteria and still menstruating regularly approximately 50% agreed to participate. There was no evidence of a difference in response rate according to mammographic pattern. None of the subjects in the present study had taken part in any of our previous studies.

*Measurements*

Socio-demographic information and epidemiologic data on risk factors for breast cancer were obtained by questionnaire.

Information on current nutrient intake was obtained with a 7-day recall form using food models to determine portion size. Subjects were then taught to maintain food records for a period of 4 consecutive days including one weekend. Subjects were given digital scales and measuring cups and spoons to weigh and measure all food and beverages consumed during the time that food records were kept. At the end of the 4-day period the dietician reviewed all records for completeness.

Venous blood was taken in both follicular and luteal phases of the menstrual cycle for measurement of oestradiol, progesterone and prolactin. Blood obtained in the luteal phase of the cycle was obtained after a 12 h fast and plasma lipids measured by the Lipid Research Clinic Core Laboratory using standard Lipid Research Clinic methods (Manual of Laboratory Operations, Lipid Research Clinics Program 1, 1982). Hormone assays were performed by the Toronto Hospitals In Common laboratory. This is a non-profit commercial laboratory with a quality control programme run by the Canadian Society of Clinical Chemists. Concentrations of oestradiol, progesterone and prolactin were measured with standard radioimmunoassays.

Each subject was weighed using a portable scale and skinfolds thickness (triceps, subscapular and iliac) was measured using Lange calipers. Percentage body fat was calculated from standard tables (Hendricks *et al.*, 1983).

*Data display and statistical methods*

'Box-plots' were used to display the distribution of scores. They are used commonly in exploratory data analysis to show as fully as possible the distribution of scores and show the distribution of the central 50% of the data values as a box with the median value shown as a cross. The range of the remaining data points is estimated by bars which extend from the box for a maximum distance 1.5 times the length of the box. Observations lying outside this estimate of the range are shown as individual points (Velleman & Hoaglin, 1981).

Proportions were compared with the $\chi^2$ statistic or Fisher's exact test when less than five were in a cell, and means with Student's $t$ test (Snedecor & Cochran, 1967). The relationship between mammographic pattern and plasma lipids was examined with simple regression, analysis of variance and analysis of covariance using the general linear model procedure. Analysis of covariance gives the same results as multiple regression. In addition, the data matrix of the final model was examined for collinearity by calculating eigenvalues using the REG procedure (SAS Users Guide: Statistics, Version 5 Edition, 1985). All analyses were performed with and without logarithmic transformation of the data. The conclusions were unchanged by logarithmic transformation and the results shown were obtained without transformation.

### Results

*Characteristics of subjects*

The two groups of women studied comprised 30 women with

**Table I** Characteristics of subjects

| Variable | Dysplasia Present | Dysplasia Absent | P value |
|---|---|---|---|
| Number | 30 | 16 | |
| Mean age (years) | 38.8±5.4 | 40.7±5.6 | 0.2 |
| Age at menarche (years) | 12.33±2.4 | 12.19±0.8 | 0.77 |
| Marital status (%) | | | |
| Married | 73 | 75 | |
| Never married | 23 | 6 | 0.05 |
| Divorced, widowed, separated | 3 | 19 | |
| Parity (%) | | | |
| 0 | 47 | 19 | |
| 1–2 | 43 | 50 | 0.08 |
| ⩾3 | 10 | 31 | |
| Familial breast cancer (%) | | | |
| 1st degree relatives | 30 | 0 | |
| Other relatives | 13 | 19 | 0.05 |
| No relatives | 57 | 81 | |
| Weight (kg) | 57.9±6.9 | 76.0±18.1 | 0.001 |
| Height (cm) | 163.4±4.7 | 164.0±7.0 | 0.7 |
| Quetelet index | 21.7±0.0002 | 28.2±0.0006 | 0.001 |
| Skin fold thickness | | | |
| Triceps | 19.5±5.5 | 26.7±5.0 | |
| Subscapular | 11.4±3.2 | 21.9±5.3 | <0.0001 |
| Illial | 10.2±5.0 | 23.0±5.4 | |

Values shown for continuous variables are means±standard deviations.

**Table II** Mean daily nutrient intake as assessed by food records

| Nutrient | | Dysplasia Present | Dysplasia Absent | P value |
|---|---|---|---|---|
| Total Energy | | 1,926±562 | 1,918±478 | 0.96 |
| Total fat | (g) | 80±28 | 84±85 | 0.5 |
| | (%) | 37±6.1 | 39±4.3 | |
| Saturated fat | (g) | 32±13.5 | 32±9.2 | 0.9 |
| | (%) | 15±3.7 | 15±2.3 | |
| Polyunsaturated fat | (g) | 13±6.1 | 14±4.9 | 0.7 |
| | (%) | 6±2.2 | 7±1.8 | |
| P/S ratio | | 0.52±0.29 | 0.47±0.15 | 0.4 |
| Protein | (g) | 75±20 | 70±20 | 0.4 |
| | (%) | 16±2.7 | 15±3.2 | |
| Carbohydrate | (g) | 203±65 | 217±67 | 0.51 |
| | (%) | 43±6.8 | 45±5.1 | |
| Cholesterol | (mg) | 400±180 | 372±145 | 0.6 |
| Alcohol | (g) | 18±24 | 5±10 | 0.01 |

Values are mean daily intake±standard deviation.

**Table III** Plasma hormones and lipids

| Variable | Dysplasia Present | Dysplasia Absent | P value |
|---|---|---|---|
| *Hormones* | | | |
| Follicular phase | | | |
| Oestradiol ($pmol\,l^{-1}$) | 465.0±296.1 | 369.7±303.5 | 0.1 |
| Progesterone ($nmol\,l^{-1}$) | 2.0±0 | 2.0±0 | 1.0 |
| Prolactin ($mg\,l^{-1}$) | 17.3±11.28 | 21.1±10.3 | 0.06 |
| Luteal phase | | | |
| Oestradiol ($pmol\,l^{-1}$) | 475.7±245.2 | 436.9±253.4 | 0.55 |
| Progesterone ($nmol\,l^{-1}$) | 39.1±24.8 | 25.7±18.4 | 0.08 |
| Prolactin ($mg\,l^{-1}$) | 19.8±15.6 | 21.2±10.3 | 0.20 |
| *Lipids* ($mmol^{-1}$) | | | |
| Cholesterol | 4.71±0.57 | 4.90±0.56 | 0.27 |
| HDL-C | 1.76±0.36 | 1.25±0.28 | 0.0001 |
| LDL-C | 2.70±0.49 | 3.34±0.59 | 0.0014 |
| VLDL-C | 0.25±0.12 | 0.32±0.14 | 0.1104 |
| Triglyceride | 0.94±0.41 | 1.38±0.5 | 0.0068 |

Values shown are means±standard deviations.

extensive mammographic dysplasia and 16 women with dysplasia occupying less than 25% of the breast. Table I shows the demographic, reproductive and anthropometric characteristics of these subjects. Compared to women with little or no dysplasia, women with extensive dysplasia were less likely to be married, more likely to be nulliparous and more likely to have first degree relatives with breast cancer. Women with dysplasia also weighed less, and as indicated by the Quetelet index and measurements of skinfolds thickness, had less body fat. The associations seen here between mammographic dysplasia and leanness, nulliparity and a family history of breast cancer have been previously observed by others (Gravelle *et al.*, 1982; de Waard *et al.*, 1984; Wolfe *et al.*, 1980).

Women with and without extensive mammographic dysplasia did not differ significantly in the proportion of former or current smokers or in the average number of hours per week spent in physical exercise (data not shown).

### *Nutrient intake*

Table II shows the nutrient intake of these subjects, as assessed by the nutrient analysis of 4-day food records. The values shown are daily averages calculated for each nutrient over the 4-day period for which records were kept. Women with and without extensive mammographic dysplasia did not differ significantly in their consumption of total calories, total fat, type of fat, protein, carbohydrate or dietary cholesterol. Women with extensive dysplasia reported greater alcohol consumption, with an average daily intake of 18 g (approximately the equivalent of two wine glasses) compared to an average intake of 5 g per day in women without dysplasia. Women with and without dysplasia were similar with respect to the intake of vitamins, including vitamin C (data not shown).

### *Plasma hormones and lipids*

Table III shows the results of the hormone assays from blood taken in both follicular and luteal phases of the menstrual cycle, and the measurement of plasma lipids determined in fasting blood samples taken in the luteal phase of the cycle. Compared to women without extensive dysplasia, women with extensive mammographic dysplasia had slightly higher levels of oestradiol in both phases of the cycle, and slightly higher levels of progesterone in the luteal phase. Differences in values for follicular phase plasma oestradiol and luteal phase plasma progesterone were close to, but did not achieve, conventional levels of statistical significance.

Total plasma cholesterol values were not significantly different between the groups, but there were substantial differences in levels of HDL-C, LDL-C and triglycerides. Compared to women without extensive dysplasia those with dysplasia had higher levels of HDL-C and lower levels of LDL-C and TG.

The plasma lipid values obtained from this group of women with extensive mammographic dysplasia were compared with the expected distribution in the population with results that are shown in Figure 1(b). Seventeen of the 30 (57%) subjects had values for HDL-C that fell above the 75th percentile for women of the same age and 11 (37%) had values of HDL-C at or above the 90th percentile. Eighteen of the 30 subjects (59%) had values for TG that were at or below the 25th percentile. The distribution of total cholesterol and LDL-C were again predominantly in the lower part of the population distribution (data not shown).

The further analyses shown in the following sections were carried out to determine if the differences in plasma lipids found between women with and without mammographic dysplasia could be explained by any of the other differences found between these groups.

*Associations between plasma lipids and other variables: univariate analysis*

Table IV shows the univariate associations between HDL-C, LDL-C and triglycerides (TG) and other variables. The variables shown were selected either because they differed between the groups of women compared or because of a previously described association with plasma lipids. In the univariate analysis, mammographic dysplasia was significantly associated with HDL-C, LDL-C and TG levels. Weight, the Quetelet index and percentage body fat were also all significantly associated with HDL-C, LDL-C and TG. A family history of breast cancer in first degree relatives was significantly associated with both HDL-C and TG. Alcohol intake was positively associated with HDL-C level but the association was not significant in this group of subjects. Neither parity nor total dietary fat intake was significantly associated in univariate analysis with any of these plasma lipids. Dietary saturated fat was significantly associated with triglyceride level, but not with HDL-C. Additional analyses failed to show any significant

**Table IV** Plasma lipids and other variables: univariate associations

| | *HDL-C* | | *LDL-C* | | *Triglycerides* | |
|---|---|---|---|---|---|---|
| | *F value* | *P* | *F value* | *P* | *F value* | *P* |
| Mammographic pattern | 23.45 | 0.0001 | 14.51 | 0.0004 | 10.14 | 0.0003 |
| Body fat % | 12.72 | 0.001 | 4.81 | 0.0001 | 23.71 | 0.0001 |
| Weight (kg) | 12.25 | 0.0011 | 0.91 | 0.344 | 17.47 | 0.0001 |
| Quetelet index | 10.3 | 0.0025 | 2.1 | 0.154 | 12.27 | 0.0011 |
| Family history of breast cancer | 8.48 | 0.0009 | 2.05 | 0.14 | 5.18 | 0.01 |
| Parity | 1.23 | 0.30 | 1.90 | 0.16 | 0.54 | 0.59 |
| Total fat (% calories) | 0.13 | 0.724 | 0.38 | 0.542 | 2.81 | 0.101 |
| Saturated fat (% calories) | 0.25 | 0.62 | 0.02 | 0.89 | 5.10 | 0.03 |
| Alcohol | 2.44 | 0.13 | 3.09 | 0.09 | 0.73 | 0.39 |

**Figure 2** Plasma levels of high density lipoprotein cholesterol (HDL-C) and triglycerides according to the presence of mammographic dysplasia and a family history of breast cancer. (Data are shown as 'box-plots'; see Methods for explanation of symbols.)

associations between plasma lipid levels and smoking, exercise or the dietary intake of carbohydrates (data not shown).

The separate influences of mammographic dysplasia and a family history of breast cancer on levels of HDL-C and TG are illustrated in Figure 2. Plasma levels of HDL-C were highest in subjects with both mammographic dysplasia and a family history of breast cancer, lowest in those with neither of these risk factors and intermediate in women with mammographic dysplasia but no family history of breast cancer. TG levels were also influenced by both of these variables but in the opposite direction.

### *Associations between plasma lipids and other variables: multiple regression analysis*

Many of the variables shown by univariate analysis to be associated with the plasma lipids are related to each other, particularly the classification of mammographic appearance and weight and percentage body fat. To assess the independent effects of these variables, we next carried out an analysis of covariance.

Several models were examined. We included in these models all of the variables shown in the single variable analysis to be significantly associated with any lipoprotein fraction, as well as any variables known as the result of other work to be associated with HDL-C, LDL-C or TG levels. The results of the final model are shown in Table V. In this model percentage body fat is included because it was more strongly associated with lipoprotein levels than any of the indices of body size, and saturated fat was included because it was significantly associated with levels of lipoprotein, while total fat and other nutrients were not. A family history of breast cancer was retained because it was independently associated with lipid levels. Parity and alcohol intake were not independently associated with lipid levels but did differ significantly between women with and without mammographic dysplasia and were included to demonstrate their lack of influence on the association of mammographic pattern with lipid levels.

In the final model shown in Table V, mammographic dysplasia and a family history of breast cancer both showed an independent and significant positive association with HDL-C level. TG level was negatively associated with a family history of breast cancer but not, after adjustment for other variables, with mammographic pattern. In addition, alcohol consumption was independently and positively associated with TG level. Neither mammographic pattern nor family history of breast cancer retained an independent association with the level of LDL-C after adjustment for the other variables shown. However, examination of the data matrix for collinearity suggested that the lack of association of LDL-C and TG with mammographic dysplasia could be due to the strong association between mammographic pattern and percentage body fat.

In the model shown, approximately 60% of the variance in HDL-C and TG was explained by the model. Mammographic pattern and a family history of breast cancer alone explained 90% of the explained variance in HDL-C level.

### Discussion

These results indicate that plasma levels of HDL-C are related to mammographic dysplasia and that levels of both HDL-C and TG are related to a familial history of breast cancer. The results also show evidence of an association between mammographic dysplasia and percentage body fat and alcohol intake. Mammographic dysplasia has previously been described as having an inverse association with weight (Gravelle *et al.*, 1985; Brisson *et al.*, 1984) and premenopausal risk of disease has also been associated with leanness (Willet *et al.*, 1985). This is in contrast to risk of postmenopausal breast cancer, where obesity appears to increase risk (see Rose (1986) for a recent review).

Although both body weight and alcohol intake are known to influence HDL-C levels, these associations did not explain the relationships observed here between HDL-C and mammographic dysplasia. Percentage body fat and mammographic dysplasia are, however, too closely related in these data to determine whether TG levels were independently associated with mammographic dysplasia.

Higher than average age- and sex-specific values of HDL-C have now been observed in two distinct groups of subjects with mammographic dysplasia and thus seem unlikely to be due to chance. The observation that both HDL-C and TG levels were related to a family history of breast cancer is based on a very small number of subjects and should therefore be regarded as preliminary. However, in the present data two risk factors for breast cancer, mammographic dysplasia and a family history of breast cancer, explained a substantial proportion of the variance in HDL-C.

Both the nature and the mechanism of the plasma lipid findings seen in association with mammographic dysplasia are presently unknown. The differences seen between women with and without extensive mammographic dysplasia both in plasma levels or HDL-C and TG and in body fat stores suggests that differences in fat metabolism exist between women with and without these radiological changes. Furthermore, differences in the quantity of fat in the breast may explain differences in the radiographic density of the breast on mammography.

The possibility cannot be excluded that the observed association of plasma lipids with mammographic dysplasia is due to hormonal influences, although previous workers and the present study have failed to find any association between levels of endogenous sex hormones and mammographic features of breast tissue (Meyer *et al.*, 1986). In the present study concentrations of plasma sex hormones were, with the exception of luteal phase prolactin, similar in women with and without mammographic dysplasia, but the number of subjects is too small to exclude possible differences of biological significance. Furthermore, in view of the reported association between body fat stores and menstruation (Frisch, 1985), the selection of women with regular menstrual cycles of defined length for this study may have contributed to our failure to find differences in hormone levels.

Plasma lipids have not been studied extensively in patients with breast cancer and the studies that have been reported

**Table V** Plasma lipids and other variables: analysis of covariance

| | *HDL-C* | | *LDL-C* | | *Triglycerides* | |
|---|---|---|---|---|---|---|
| | *F value* | *P* | *F value* | *P* | *F value* | *P* |
| Mammographic pattern | 10.3 | 0.003 | 0.01 | 0.94 | 0.77 | 0.39 |
| Body fat % | 0.68 | 0.41 | 4.80 | 0.04 | 5.83 | 0.007 |
| Family history of breast cancer | 6.83 | 0.003 | 0.07 | 0.93 | 6.44 | 0.004 |
| Parity | 1.11 | 0.34 | 0.32 | 0.73 | 0.85 | 0.44 |
| Saturated fat (% calories) | 1.19 | 0.28 | 0.67 | 0.42 | 8.41 | 0.007 |
| Alcohol | 0.05 | 0.82 | 1.84 | 0.18 | 2.24 | 0.15 |
| $R^2$ | 0.60 | | 0.40 | | 0.63 | |

are inconsistent in their results. Rossner and Wallgren (1984) studied 23 post-menopausal women after surgical removal of apparently localised breast cancer, and found that plasma HDL-C was significantly higher in cancer patients compared to 35 healthy controls. Miller and Erf (1956) studied eight women after apparently curative surgical resection of breast cancer and found significantly higher levels of HDL-C than in blood donors free of breast cancer. The same authors found significantly lower levels of HDL-C in women with metastatic breast compared to controls. Feldman and Carter (1971) studied post-menopausal women with metastatic breast cancer and found significantly lower HDL-C levels in cancer patients. Barclay *et al.* (1970, 1975) also reported a reduction in HDL-C in women from whom blood was obtained in the presence of either an unresected breast tumour or of metastatic disease. Bani *et al.* (1986) studied pre- and post-menopausal women before resection of breast tumours and found a significantly lower level of HDL-C in women with breast cancer than in controls.

In all of these reports the size of groups studied has been small, and potential confounding variables such as age, weight, menopausal status and alcohol intake have not been taken into account. None the less, the reported studies all suggest that breast cancer is associated with an abnormality of HDL-C levels. The very limited evidence available on patients with and without resected breast cancer suggests that plasma levels of HDL-C may be influenced by the presence of tumour.

Furthermore, plasma levels of HDL-C are influenced by other factors known or suspected to affect breast cancer risk, including female sex hormones (Srinivasan *et al.*, 1985; Wahl *et al.*, 1983), parity (van Stiphout *et al.*, 1987), dietary fat (Jones *et al.*, 1987; Goodwin & Boyd, 1987) and alcohol (Graham, 1987; Williams *et al.*, 1985).

Interest in the relationship of HDL-C to disease has to date been focused primarily on its protective role in coronary heart disease (Miller & Miller, 1975), a disease from which women have some protection that appears to be related at least in part to their higher levels of HDL-C. The results shown here raise the possibility that HDL-C may be related to breast cancer risk, a disorder to which women in the Western world are especially susceptible, and suggest that further investigation is warranted of the role of plasma lipids in relation to this disease.

We thank Dr P.W. Connelly, Director, Lipid Research Clinic Core Laboratory, who performed the lipid assays reported here, and Dr S. Tilak, Director, Hospitals In Common Laboratory, Toronto, where hormone assays were performed. We also thank Dr I. Simor for classifying mammograms at Mount Sinai hospital.

### References

BANI, I.A., WILLIAMS, C.M., BOULTER, P.S. & DICKERSON, J.W.T. (1986). Plasma lipids and prolactin in patients with breast cancer. *Br. J. Cancer*, **54**, 439.

BARCLAY, M. & SKIPSKI, V.P. (1975). Lipoproteins in relation to cancer. *Prog. Biochem. Pharmacol.*, **10**, 76.

BARCLAY, M., SKIPSKI, V.P., TEREBUS-KEKISH, O. *et al.* (1970). Effects of cancer upon high density and other lipoproteins. *Cancer Res.*, **30**, 2420.

BOYD, N.F., O'SULLIVAN, B., CAMPBELL, J.E. and 4 others (1982). Mammographic signs as risk factors for breast cancer. *Br. J. Cancer*, **45**, 185.

BRISSON, J., MERLETTI, F., SADOWSKI, N.L. *et al.* (1982). Mammographic parenchymal patterns of the breast and breast cancer risk. *Am. J. Epidemiol.*, **115**, 428.

BRISSON, J., MORRISON, A.S., KOPANS, D.B. *et al.* (1984). Height and weight, mammographic features of breast tissue and breast cancer risk. *Am. J. Epidemiol.*, **119**, 371.

DE WAARD, F., ROMBACH, J.J., COLLETTE, H.J.A. & SLOTBOOM, B. (1984). Breast cancer risk associated with reproductive factors and breast parenchymal patterns. *J. Natl Cancer Inst.*, **72**, 1277.

FELDMAN, E.B. & CARTER, A.C. (1971). Circulating lipids and lipoproteins in women with metastatic breast carcinoma. *J. Clin. Endocrinol.*, **33**, 8.

FRISCH, R. (1985). Fatness, menarche, and female fertility. *Perspect. Biol. Med.*, **28**, 611.

GOODWIN, P. & BOYD, N.F. (1987). A critical appraisal of the evidence that dietary fat intake is related to breast cancer risk in humans. *J. Natl Cancer Inst.*, **79**, 473.

GOODWIN, P.J. & BOYD, N.F. (1988). Mammographic parenchymal pattern and breast cancer: a critical appraisal of the evidence. *Am. J. Epidemiol.*, **127**, 1097.

GRAHAM, S. (1987). Alcohol and breast cancer risk, Editorial. *N. Engl. J. Med.*, **316**, 1211.

GRAVELLE, I.H., BULSTRODE, J.C., BULBROOK, R.D. *et al.* (1982). The relation between radiologic patterns of the breast and weight and height. *Br. J. Radiol.*, **55**, 23.

HENDRICKS, S., KROETSCH, D., NIELSON, H., SOUCY, P. & VERDIER, P. (1983). Guide for anthropometric measurement of Canadian adults. Ross Laboratories Ltd., Montreal.

JONES, D.Y., JUDD, J.T., TAYLOR, P.R., CAMPBELL, W.S. & NAIR, P.P. (1987). Influence of caloric contribution and saturation of dietary fat on plasma lipids in premenopausal women. *Am. J. Clin. Nutr.*, **45**, 1451.

LEE-HAN, H., COUSINS, M., BEATON, M. *et al.* (1988). Measurement of compliance in a randomized clinical trial of dietary fat reduction in patients with breast dysplasia: relationship between nutrients and plasma lipids. *Am. J. Clin. Nutr.*, **48**, 575.

LIPID RESEARCH CLINICS PROGRAM EPIDEMIOLOGY COMMITTEE (1979). Plasma lipid distribution in selected North American populations. *Circulation*, **60**, 427.

MILLER, B.J. & ERF, L. (1956). The serum proteins and lipoproteins in patients with carcinoma and in subjects free of recurrence. *Surg. Gynecol. Obstet.*, **102**, 487.

MILLER, G.J. & MILLER, N.E. (1975). Plasma-high-density-lipoprotein concentration and development of ischaemic heart disease. *Lancet*, **i**, 16.

ROSE, D.P. (1986). Dietary factors and breast cancer. *Cancer Surv.*, **5**, 671.

ROSSNER, S. & WALLGREN, A. (1984). Serum lipoproteins and proteins after breast cancer surgery and effects of tamoxifen. *Atherosclerosis*, **52**, 339.

SAFTLAS, A.F. & SZKLO, M. (1987). Mammographic parenchymal patterns and breast cancer risk. *Epidemiol. Rev.*, **9**, 146.

SNEDECOR, G.W. & COCHRAN, W.G. (1967). *Statistical Methods*, 6th ed. Iowa State University Press: Ames, Iowa.

SRINIVASAN, S.R., SUNDARAM, G.S., WILLIAMSON, G.D., WEBBER, L.S. & BERENSON, G.S. (1985). Serum lipoproteins and endogenous sex hormones in early life: observations in children with different lipoprotein profiles. *Metabolism*, **34**, 861.

VAN STIPHOUT, W.A.H., HOFMAN, A. & DE BRUIJN, A.M. (1987). Serum lipids in young women before, during, and after pregnancy. *Am. J. Epidemiol.*, **126**, 922.

VELLEMAN, P.F. & HOAGLIN, D.C. (1981). *Applications, Basics and Computing of Exploratory Data Analysis*. Duxbury Press: Boston.

WAHL, P., WALDEN, C., KNOPP, R. *et al.* (1983). Effect of estrogen/progestin potency on lipid/lipoprotein cholesterol. *N. Engl. J. Med.*, **308**, 862.

WILLET, W.C., BROWNE, M.L., BAIN, C. *et al.* (1985). Relative weight and risk of breast cancer among premenopausal women. *Am. J. Epidemiol.*, **122**, 731.

WILLIAMS, P.T., KRAUSS, R.M., WOOD, P.D., ALBERS, J.J., DREON, D. & ELLSWORTH, N. (1985). Association of diet and alcohol intake with high density lipoprotein subclasses. *Metabolism*, **34**, 524.

WOLFE, J.N., ALBERT, S., BELLE, S. *et al.* (1980). Familial influences on breast parenchymal patterns. *Cancer*, **46**, 2433.

WOLFE, J.N., SAFTLAS, A.F. & SALANE, M. (1987). Mammographic parenchymal patterns and quantitative evaluation of mammographic densities: a case control study. *Am. J. Roentgenol.*, **148**, 1087.